# Older Adults’ Persisting Beliefs About the Death Panel Myth and Advance Directives

**DOI:** 10.1093/geroni/igaa057.223

**Published:** 2020-12-16

**Authors:** Allyson Coldiron, Lauren Bennett-Leleux, Diamond Lee, Lucas Childers, Rachel Donnell, Alexandra Sandlin, Arthur Marsden, Michael Barnett

**Affiliations:** 1 The University of Texas at Tyler, Tyler, Texas, United States; 2 The Memory Assessment and Research Center, The University of Texas at Tyler, Tyler, Texas, United States; 3 University of Texas at Tyler, Tool, Texas, United States; 4 Syracuse University, Syracuse, New York, United States

## Abstract

The political debate preceding passage of the Affordable Care Act included controversy over a bill that some claimed would establish a “death panel” to judge if older adults were worthy of receiving medical care. This claim was false, as the bill would instead incentivize physicians to inform Medicare patients about advanced directives: legal documentation of one’s end-of-life preferences. However, the death panel myth led to the removal of this bill from the Affordable Care Act, and a poll five years later found 41% of Americans still believed in the death panel myth. We investigated the effects believing in this myth had on older adults, hypothesizing that those who believed in the myth would have lower advance directive completion rates and more negative attitudes towards advanced directives. Community-dwelling older adults aged 65 to 102 years (N = 182) in a large city in the southern United States completed an interview survey. No relationship was found between belief in the death panel myth and advanced directive completion; however, older adults who believed in the myth had lower perceived need for advanced directives than those who did not. Surprisingly, 47.1% of older adults who believed in the myth also supported incentivizing doctors to inform patients about advanced directives, suggesting that many older adults who believe in the myth do not know that the controversial bill was about advanced directives. Results suggest that the death panel myth may have long-lasting effects, specifically persistent distrust about policies promoting advance directives.

